# Use of indocyanine green near-infrared lymphography to detect sentinel lymph nodes in a dog with a malignant insulinoma: a case report

**DOI:** 10.3389/fvets.2023.1178454

**Published:** 2023-04-27

**Authors:** Mirja Christine Nolff, Renate Dennler, Matthias Dennler

**Affiliations:** ^1^Clinic für Small Animal Surgery, Vetsuisse Faculty, University of Zurich, Zurich, Switzerland; ^2^Clinic of Diagnostic Imaging, Department of Clinical Services, Vetsuisse Faculty, University of Zurich, Zurich, Switzerland; ^3^Kleintierklinik Dennler, Affoltern am Albis, Switzerland

**Keywords:** functional neuroendocrine pancreatic tumor, pancreatic lymph node mapping, sentinel node mapping, staging malignant melanoma, lymphadenectomy

## Abstract

Malignant insulinoma is the most common type of neuroendocrine tumor found in the pancreas of dogs. Canine insulinoma displays malignant behavior with a high rate of metastasis. The most common sites of metastases are the draining lymph nodes, which are also the primary location sites for the recurrence of functional disease. However, identifying metastatic nodes can often be complicated, as the pancreas is drained by numerous lymphatic centers, and clinical enlargement or structural changes may not always be present in metastatic nodes. Additionally, unaltered nodes are frequently small (a few millimeters) and can be hard to distinguish from the surrounding tissues. Therefore, lymphadenectomy is generally recommended for affected dogs. Unlike in human medicine, there are currently no established strategies for lymph node resection in dogs with malignant insulinoma. This report presents a technique for identifying and removing sentinel nodes using indocyanine green and near-infrared lymphography (NIRFL) during surgery. A total of six sentinel nodes were detected and resected with this method. This technique could provide a more structured approach for lymph node resection in affected dogs and potentially in humans in the future. However, its therapeutic benefits must be evaluated in a larger cohort of cases.

## 1. Introduction

Canine insulinoma is a tumor that develops from the beta cells of the pancreas and is the most common neoplasm affecting the endocrine pancreas in dogs (1–6). Unlike human pancreatic tumors, most canine insulinomas are mostly both malignant and functional (7, 8). Surgical resection is the recommended treatment option for affected dogs with a median survival time (MST) ranging between 372 and 785 days (1–4, 9–11).

Gross metastatic disease is detectable in up to 50% of dogs with insulinoma at the time of initial diagnosis, with lymph nodes and the liver being the most commonly affected sites (3, 4, 10, 11). As insulinoma tumors are usually functional, removal of all potential metastatic lesions is recommended whenever possible, as persistent postoperative hypoglycemia is a major negative prognostic factor. (2–4, 10–12). Unfortunately, clinical and pathological staging do not always correlate well, as lymph nodes with metastases may not display abnormal size or texture (12–15). Consequently, removing only a single altered node is not sufficient for precise staging and carries the risk of leaving functional tissue behind.

Given the challenges associated with identifying metastatic lymph nodes in canine insulinoma, it appears reasonable to focus on the draining lymph nodes of the pancreas region, known as the sentinel nodes, rather than attempting to identify individual nodes. However, the complex drainage pattern of the pancreas and the poor visibility of small nodes make this approach difficult. Based on Baum's anatomy of the canine lymphatic system, numerous lymph nodes potentially drain the pancreas, including 3 left hepatic nodes, 1–5 right hepatic nodes, 1–5 splenic nodes, a duodenal lymph node, and two jejunal nodes, resulting in 6–16 possible sentinel lymph nodes (SLNs) (16).

Sentinel node mapping using indocyanine green (ICG) and near-infrared fluorescence (NIR-F) offers a potential alternative approach for identifying and removing early metastatic lymph nodes in affected dogs. In human medicine, SLN mapping using ICG and NIR-F has been mainly used to detect the intestinal nodes in patients with colonic or gastric cancer, with varying success rates (17–19). To date, no reports of intestinal SLN mapping exist for dogs (20). Previous attempts to map the lymphatics of pancreatic neoplasia in humans using methylene blue have been reported to show insufficient performance (21, 22); however, studies or case reports on NIR-F lymphography in pancreatic tumors is currently lacking in humans and dogs.

This case described the first application of ICG NIRF to map the lymphatics in a dog with naturally occurring malignant insulinomas.

## 2. Case description

In April 2022, an 11.5-year-old intact female Border Collie was presented after experiencing a single generalized seizure. In May 2022, the dog experienced a second seizure episode during which the owner measured the dog's glucose which was 1.5 mmol/l. Apart from these episodes, the dog was considered healthy and had not shown any signs of illness in the past year. In addition, the dog was also known to have biceps tendinopathy.

### 2.1. Clinical findings

During the first presentation, the dog weighed 17.4 kg (BCS 5) and appeared clinically unremarkable, except for a slight bradycardia (heart rate: 54/BPM) and a 2/6 left-sided systolic heart murmur. Multiple fractured teeth were also observed.

### 2.2. Diagnostic assessment

**Figure 1:**
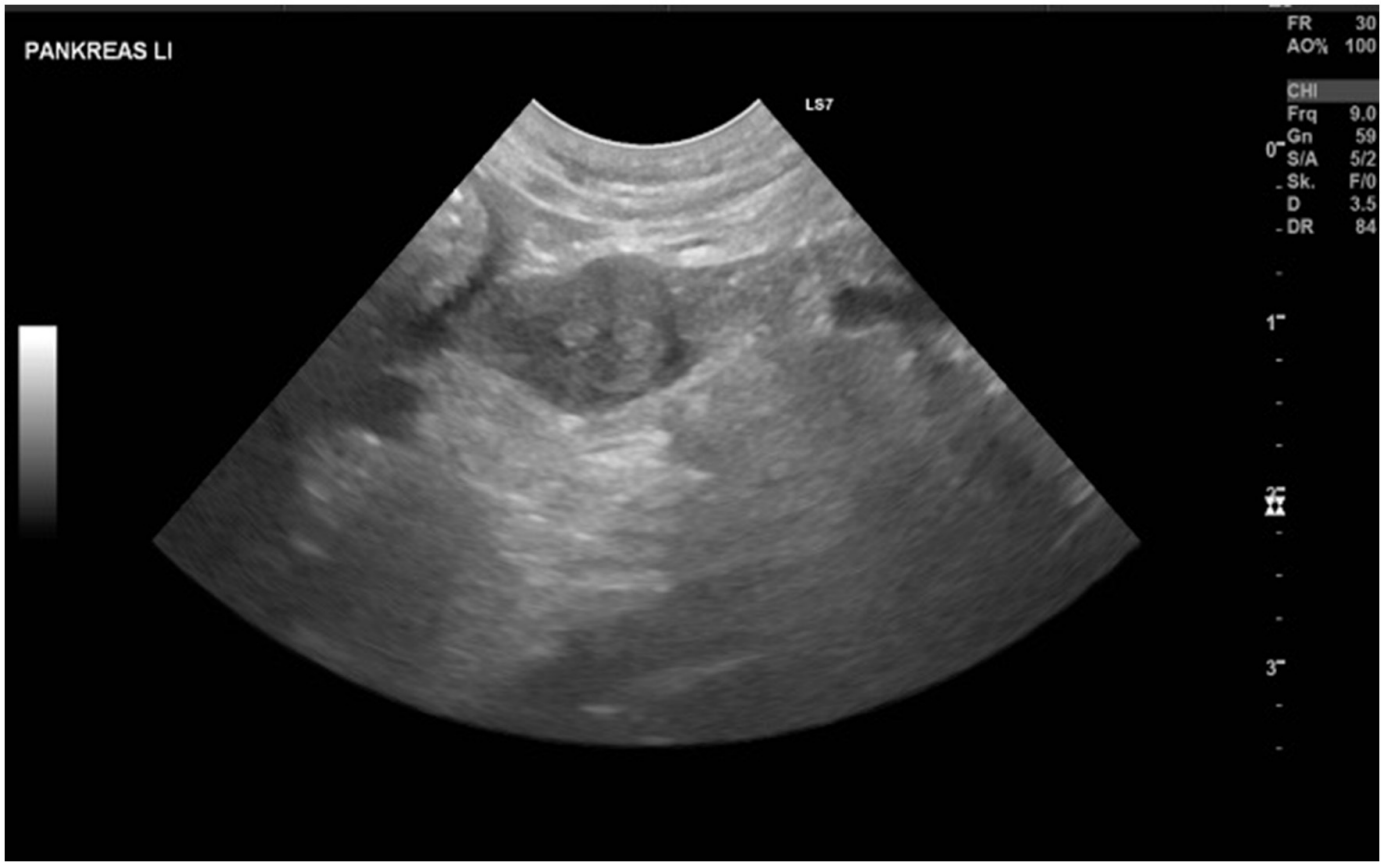
Showing the lesion as well as the enlarged lymph node detected at the initial ultrasound examination.

Blood work revealed a moderate increase in liver enzymes (GPT 734 U/L, reference range: 17–78; ALP 157 U/L, reference range: 13–83) and mild thrombocytopenia (PLT 101 E3μl, reference range: 148–484) but was otherwise normal. Blood glucose at presentation was 4.2 mmol/L (reference range: 4.2–7.1). An abdominal ultrasound revealed a mildly enlarged and rounded liver with multiple small nodular areas with mixed echogenicity, sludge accumulation within the gallbladder, a small (19 mm diameter) heterogeneous cavitary lesion at the tail of the spleen, and a 9-mm sharply circumscribed, highly vascularized nodular structure in the left pancreatic branch. A single enlarged (7 mm) mesenteric lymph node with a heterogeneous echotexture in the cranial abdomen was also identified, along with multiple small mural cystic lesions and minimal intraluminal fluid in the uterus. Figure 1 shows the ultrasonographic findings of the pancreas and the enlarged lymph node. The ventrodorsal and right lateral thoracic radiographs were unremarkable.

The dog was then presented for work up at the Clinic for Small Animal Surgery, Vetsuisse Faculty, Zürich University. Helical CT scans were performed using a Philips Brilliance16 scanner (Philips AG, Zurich, Switzerland) for the thorax and abdomen, which were reconstructed using a soft tissue, bone, and lung algorithm for the thorax and a soft tissue and bone algorithm for the abdomen. A triple-phase CT scan of the abdomen was timed with bolus tracking in the descending aorta and reconstructed using a soft tissue algorithm, with 2 ml/kg Accupaque 350 (GE Healthcare, Glattbrugg, Switzerland) administered through a power injector (Accutron CT-D Medtron Injector, SMD Medical Trade GmbH, Salenstein, Switzerland). The images were reviewed on a workstation (IntelliSpace PACS 4.4 Radiology, Philips AG, Zurich, Switzerland), revealing a well-defined focal, round, soft tissue-attenuated nodule (1 cm in diameter) located in the caudal third of the left pancreatic branch. The nodule exhibited marked contrast enhancement during the arterial phase, with attenuation differing from normal pancreatic tissue, which equalized in the venous phase (early wash in late wash out). A lymph node (considered hepatic) was found to be mildly enlarged but showed normal contrast uptake. An intraparenchymal nodule measuring 2 cm with rim enhancement was detected in the body of the spleen. The liver presented with multiple ill-defined, hypoattenuating areas in all stages of contrast distribution. A small amount of mineral attenuating material occurred in the gallbladder. The uterus contained a single oval-shaped lesion in the left horn and minimal fluid.

Based on the imaging results, the suspected diagnoses were malignant insulinoma (with potential differential diagnoses of carcinoma) with concurrent lymphadenopathy (although a metastatic lesion was considered less likely), nodular hepatopathy (unspecific), a splenic lesion (hematoma, hyperplasia, or neoplasia), and an endometrial cyst.

Repeat blood glucose testing revealed a glucose level of 5.6 mmol/L. Despite a high suspicion of insulinoma, no additional insulin analysis was performed since the dog had euglycemia at presentation, and insulin concentration is only reliable during hypoglycemia (< 3.3 mmol/L). Surgery was recommended and scheduled for 10 days after the workup. Initial therapy involved administering 2 mg/kg of oral prednisone one time daily until surgery.

### 2.3. Intervention

Anesthesia was induced using fentanyl (3 μg/kg IV) and alfaxalone (1 mg/kg IV Alfaxalon Multidose, Dr. Graeub AG, Bern) and maintained after endotracheal intubation using sevoflurane (Sevorane^®^, AbbVie AG, Cham) delivered in oxygen. During surgery, analgesia was maintained by administering fentanyl (10 μg/kg/h, Fentanyl Sintetica, Sintetica, Mendrisio, CRI). In addition, the dog received lidocaine (30 μg/kg/h, Lidocaine HCL Bichsel, Bichsel AG, Interlaken, CRI) and dobutamine (2.5 μg/kg/min, Dobutrex^®^, Teva AG, Rapperswil). At induction, the blood glucose level was 3.7 mmol/L.

**Figure 2:**
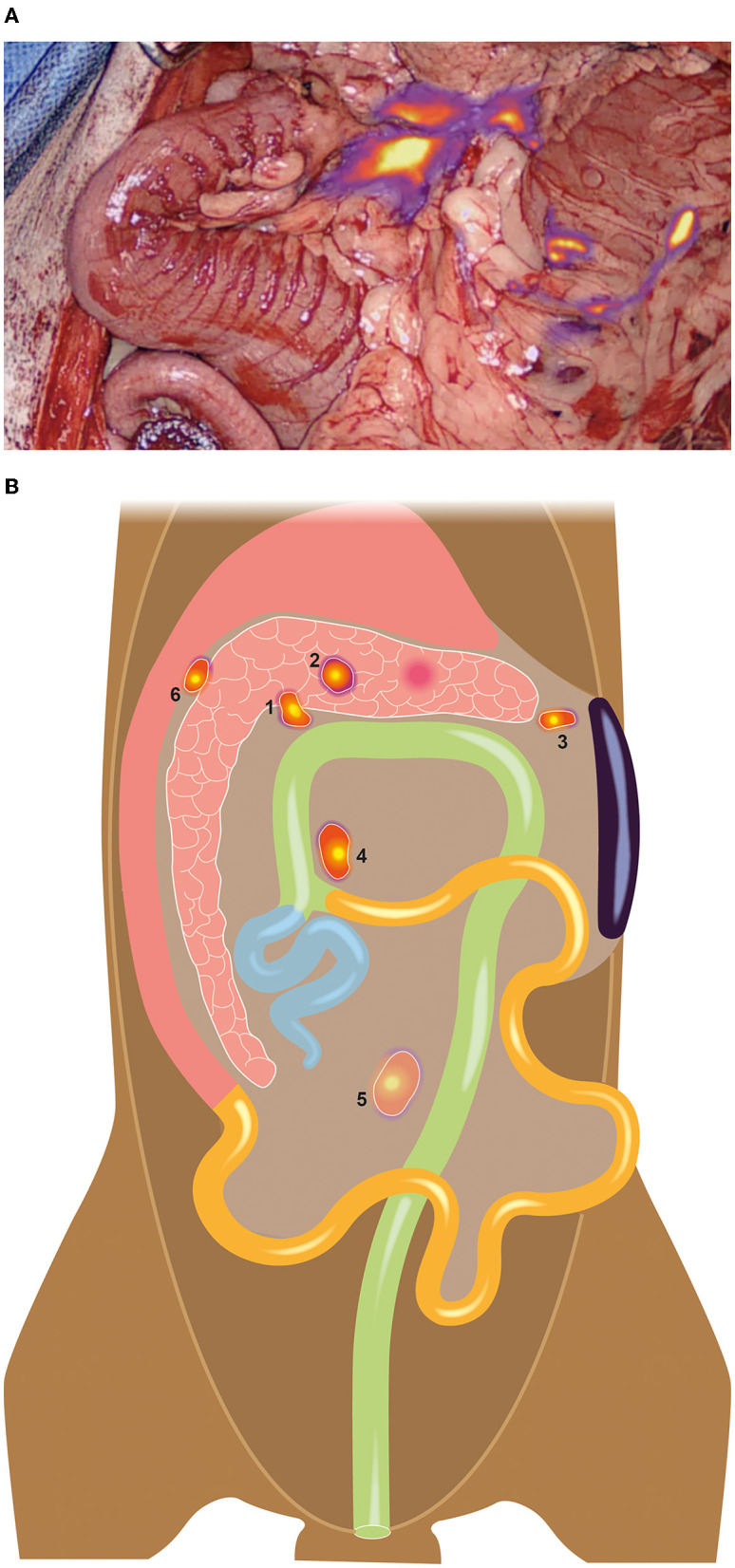
A total of six draining nodes was detected using NIR lymphography **(A)**, three of which were not palpable or visible without NIR guidance during surgery. **(B)** Schematic illustration of the detected nodes: 1 = Right hepatic lymph node, 2= left hepatic lymph node, 3= splenic lymph node, 4= right colic lymph node, 5= jejunal lymph node, 6= duodenal lymph node.

After performing a standard coeliotomy, the mass in the pancreas was visualized, and a complete inspection of the abdomen was conducted. Apart from the jejunal (1.5 × 1.5 × 0.5 cm) and right colic lymph nodes (1.5 × 1 × 0.5 cm), one additional node was identified between the pancreas and the stomach (the duodenal lymph node, 1.4 × 0.8 × 0.3 cm). Subsequently, 0.2 ml of ICG (2.5 mg/kg, Verdye, Diagnostic Green GmbH, Kirchheim) was injected into the tumor. Lymphatics became visible within seconds (Supplementary Video 1, Visionsense, VS3 Iridium, Medtronic, Switzerland), resulting in the identification of a total of six draining nodes, including the three nodes previously identified prior to injection, as well as the right (0.6 × 0.4 × 0.4 cm) and left hepatic nodes (0.2 × 0.2 × 0.2 cm) and a splenic node (0.5 × 0.5 × 0.5 cm) (please refer to Figure 2).

All nodes were removed using a ligature (LigaSureTM Small Jaw Instrument, Medtronic, Switzerland). The ligature also removed the pancreatic mass through an en-bloc resection of the left branch of the pancreas. After completion of the lymphadenectomies, blood glucose was measured at 4.1 mmol/L. Blood glucose levels were recorded again after partial pancreatectomy and were found to be 5.9 mmol/L and 6 mmol/L at the end of surgery. Liver biopsies were taken from the left and central lobes of the liver, where nodular areas were visible. Finally, a splenectomy and an ovariohysterectomy were performed. The total surgical time was 68 m (the total anesthesia time was 145 m).

Before wound closure, 2 mg/kg ropivacaine (total volume 4.3 ml; Ropivacaine Sintetica, Sintetica, Mendrisio) was administered into the abdomen. Postoperative analgesia was maintained using methadone 0.1 mg/kg IV (Methadon Streuli, Streuli Pharma AG, Uznach) q4 h for 24 h and metamizole (Minalgin, Streuli Pharma AG, Uznach) 25 mg/kg IV q8 h for 24 h, followed by oral administration for 7 days. Prednisone was gradually tapered by administering 0–5 mg/kg every other day for two additional doses. The dog remained hypoglycemic after surgery and was stable enough to be discharged the following day.

Histopathologic examination confirmed the presence of a well-demarcated and densely cellular neoplasm with a diameter of 15 mm, surrounded by a fibrous capsule invaded by neoplastic cells. The mitotic rate was low (1 in 10 HPF), and immunohistochemistry was negative for CAM-5.2 and strongly positive for anti-insulin, leading to a final diagnosis of malignant insulinoma. No signs of lymph node metastasis were detected (Stage I). The splenic lesion was diagnosed as nodular hyperplasia and the liver lesions as vacuolar hepatopathy.

### 2.4. Follow-up and outcome

**Figure 3:**
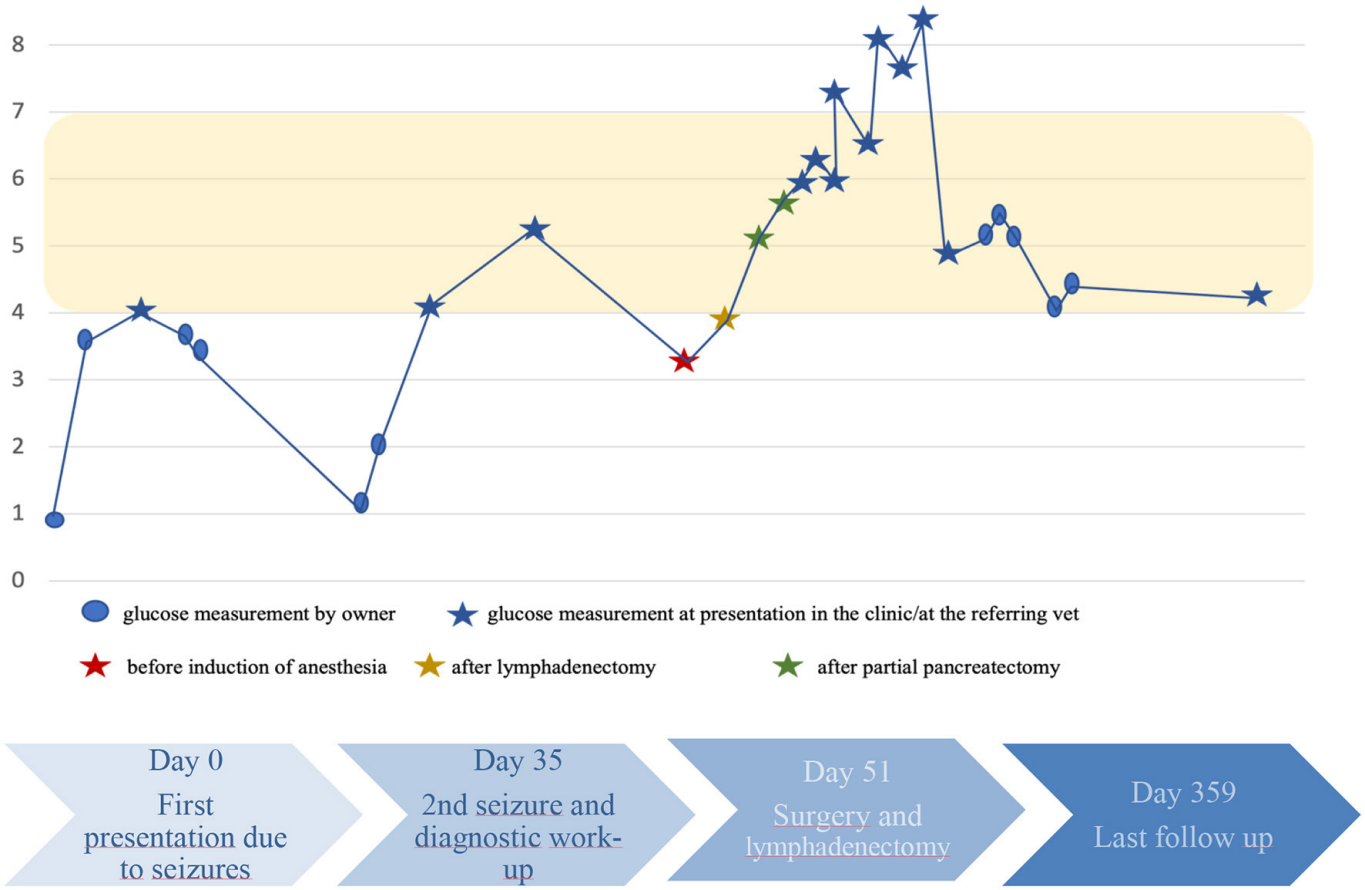
^*^Yellow box marks the reference range.

The dog's recovery was unremarkable, and up until the time of drafting the manuscript, no further seizures or hypoglycemic episodes were detected. A timeline with progression of the case is depicted in Figure 3.

## 3. Discussion

While lymph node resection is standardized in people with pancreatic carcinoma and involves the resection of 12 lymph node stations in the head and three stations in tumors of the tail or body of the pancreas (23–25), there is currently no established approach for lymph node resection in dogs with malignant pancreatic neoplasia.

The studies currently available on dogs with malignant insulinoma mostly documented the resection of grossly enlarged or “visible” nodes (with and without control of the impact on hypoglycemia) but fail to provide information on the number of nodes resected per animal (1–3, 9, 10). This approach appears overly simplistic, given the relatively complex drainage pattern of the canine pancreas and the potentially large number of SLNs.

This is especially true considering the fact that the clinical stage based on imaging (e.g., “enlarged” or structurally altered nodes in CT or ultrasound) does not correspond well to the histopathological stage. Cleland et al. (2) compared the clinical and histopathological stages in affected dogs in 2020 and documented significant discrepancies, with 25% of dogs at each stage 1, 2, 3 or with no imaging of the signs of a tumor, translating to dogs with stage I disease in 45% of cases, stage II in 24% of cases, and stage III in 31% of cases based on histology (2). Notably, nodes were only resected in 55% of cases, leaving the question of how many dogs were in stage I at the end of the study.

In another study performed by Buishand et al. (1), the sensitivity of CT to detect nodal metastasis was evaluated at 67% (1). Unfortunately, the authors failed to provide information on the number of lymph nodes that were considered normal/non-detectable in the CT that were metastatic, as these were not resected. Similar findings have been published for human pancreatic carcinoma. Cesmebasi et al. (26) found that it is problematic to identify metastatic nodes in affected patients. In MRI and surgery, 21% of their metastatic nodes were normal in size and texture (26).

In human medicine, routine lymphadenectomy is an important component in the treatment of pancreatic adenocarcinomas, but the extent of lymphadenectomy is still a topic of debate (23–27). However, a standard protocol has been widely established for pancreatic adenocarcinoma, which involves the resection of 12 or 3 lymph node stations in the head or tail/body of the pancreas, respectively (25). Extended lymphadenectomies involving the removal of more lymph nodes failed to demonstrate superior outcomes but were associated with higher morbidity for the patients (23–27).

In humans with pancreatic neuroendocrine tumors (P-NET), the current recommendation is to resect lymph nodes only in aggressive functional tumors with negative prognostic expectations or tumors that are bigger than 1–3 cm in size (28–32). Several studies have investigated the impact of nodal resections in P-NET and documented that the metastatic rate of tumors was significantly underrated if fewer than six or eight nodes were resected (31, 32). Given the malignant nature of canine insulinoma, it is most likely to fall under the subgroup of aggressive P-Net, where regional lymphadenectomy is recommended. Zhang et al. (32) conducted one of the largest studies on this topic in humans, reporting a lymph node metastatic rate of 21% and the benefit of lymphadenectomy when at least six nodes were removed (32). Wu et al. (31) found that patients with aggressive P-NET (>2 cm, Ki-67 Y3%, located in the pancreatic head) had a higher therapeutic index when at least eight nodes were removed (31). Extended radical lymphadenectomies did not appear to provide any additional value and were thus not recommended (28–32). In summary, for aggressive P-NET, moderate resections of the regional draining nodes are recommended and likely to improve outcomes in humans. As malignant insulinoma in dogs shows aggressive behavior (7), with nodal metastatic rates of 70–95%, a similar approach is likely to be reasonable.

Sentinel node mapping could help identify regional draining nodes and therefore offer a more structured approach than resecting enlarged nodes in dogs. However, early attempts in humans with pancreatic carcinoma to map sentinel nodes using methylene blue were not convincing due to low detection rates and high false-negative rates (21, 22). As methylene blue is one of the least sensitive options for nodal mapping (20), caution should be exercised when interpreting these results, and further validation of the usefulness of other mapping techniques is warranted.

In the presented case, we successfully performed lymphography on a dog with a malignant insulinoma located in the pancreas using ICG. We were able to detect and remove eight sentinel nodes, six of which would not have been detectable through visualization or palpation during surgery. Based on our findings, we concluded that ICG NIRF is generally feasible in dogs with insulinoma and allows for easy identification of small, non-altered nodes. The technique might offer a more structured approach for easy identification of small, non-altered nodes. This technique may offer a more structured approach to nodal resection in dogs with malignant insulinoma in the future. However, further studies are necessary to evaluate the performance and clinical impact of this technique, including the detection rate and false-negative rate.

## Data availability statement

The original contributions presented in the study are included in the article/Supplementary material, further inquiries can be directed to the corresponding author.

## Ethics statement

Ethical review and approval was not required for the animal study because ICG Lymphography is now a routine procedure in our facility, that is routinely used in our cases due to medical/treatment reasons. As this is merely a retrospective description of mapping of a new tumor type in a single dog that was done due to medical reasons it does not require an animal license. Written informed consent was obtained from the owners for the participation of their animals in this study.

## Author contributions

The original draft of the manuscript was written by MN. MD and RD also contributed to the initial draft and provided revisions for the above manuscript. All authors contributed to the article and approved the submitted version.

## Supplementary material

Supplementary Video 1 ICG Lymphography.
